# Comprehensive Analysis of the Real Lifestyles of T1D Patients for the Purpose of Designing a Personalized Counselor for Prandial Insulin Dosing

**DOI:** 10.3390/nu11051148

**Published:** 2019-05-23

**Authors:** Katerina Stechova, Jan Hlubik, Pavlina Pithova, Petr Cikl, Lenka Lhotska

**Affiliations:** 1 Department of Internal Medicine, University Hospital Motol, V Uvalu 84, 15006 Prague 5—Motol, Czech Republic; pavlina.pithova@fnmotol.cz; 2 The Czech Institute of Informatics, Robotics and Cybernetics, Czech Technical University in Prague, Jugoslavskych partyzanu 1580/3, 160 00 Prague, Czech Republic; hlubikjan@post.cz (J.H.); lenka.lhotska@cvut.cz (L.L.); 3 Fitsport Complex Inc., Polní 1006/11, 664 91 Ivancice, Czech Republic; petrcikl@seznam.cz; 4 Faculty of Biomedical Engineering, Czech Technical University in Prague, nam. Sitna 3105, 272 01 Kladno, Czech Republic

**Keywords:** carbohydrate counting, diet, mobile application, obesity, overweight, postprandial glycaemia, prandial insulin bolus, type 1 diabetes

## Abstract

Post-prandial hyperglycemia is still a challenging issue in intensified insulin therapy. Data of 35 T1D patients during a four-week period were analyzed: RT-CGM (real time continuous glucose monitoring) record, insulin doses, diet (including meal photos), energy expenditure, and other relevant conditions. Patients made significant errors in carbohydrate counting (in 56% of cooked and 44% of noncooked meals), which resulted in inadequate insulin doses. Subsequently, a mobile application was programmed to provide individualized advice on prandial insulin dose. When using the application, a patient chooses only the type of categorized situation (e.g., meals with other relevant data) without carbohydrates counting. The application significantly improved postprandial glycemia as normoglycemia was reached in 95/105 testing sessions. Other important findings of the study include: A high intake of saturated fat (median: 162% of recommended intake); a low intake of fiber and vitamin C (median: 42% and 37%, respectively, of recommended intake); an increase in overweight/obesity status (according to body fat measurement), especially in women (median of body fat: 30%); and low physical activity (in 16/35 patients). The proposed individualized approach without carbohydrate counting may help reach postprandial normoglycemia but it is necessary to pay attention to the lifestyle habits of T1D patients too.

## 1. Introduction

The therapeutic targets for patients with type 1 diabetes are clearly defined: Achieve (near) normoglycemia without severe hypoglycemic events, maintain other biochemical parameters within the normal range (lipids, etc.), and maintain or lower blood pressure and anthropometric parameters (body weight, waist/hip ratio). For other important therapeutic goals, optimal physical fitness and an overall good quality of life should be considered [1]. Despite all the modern treatment modalities, reality is often far from the optimal situation described.

The first hybrid closed-loop system with official FDA approval is now available in some countries. In hybrid closed-loop systems (approved or not yet), basal doses are driven automatically according to continuous glucose monitoring (CGM) data to maintain glycemia within the target range. Patients should apply manual boluses by using a bolus calculator and CGM data but reaching post-meal normoglycemia without subsequent hyperglycemia or hypoglycemia due to insulin under-correction or over-correction, respectively, is difficult even for the most advanced algorithms [2].

The basic input variables of the algorithms of current bolus calculators are actual glycemia, the amount of active insulin and the carbohydrate content of the meal that is the value calculated (estimated) by the patient. Insulin sensitivity factor (ISF), carbohydrate to insulin ratio (CIR), target glycemia, and time of active insulin are pre-set parameters [3].

To facilitate patients treated for diabetes with their day-to-day decisions, we started a partnership with colleagues at the Czech Technical University to develop a mobile phone application that acts as a personalized counselor, especially for helping the user make the optimal choice of prandial insulin dose. This tool was intended to be fully personalized and easy-to-use. We originally designed the application only for patients treated with an insulin pump (continuous insulin infusion regime, CSII) but later on, we extended the pilot version to patients treated with the MDI (multiple daily insulin injection) regimen as well.

The primary goal of this proof-of-concept study was to test the effectiveness of an application prototype to reduce postprandial hyperglycemia, so the primary outcome of our study was the frequency of postprandial normoglycemia (without using vs. by using the application). In order to create an application prototype, a detailed analysis of the lifestyles and decision-making strategies of patients with type 1 diabetes regarding prandial insulin dose was provided. We specifically focused on three areas. The first area was eating habits, diet composition, and carbohydrate counting. The second area was an analysis of whether patients consider their physical activity (previous or planned) in their bolus decision (and how), and finally, whether the patients take into account the presence of a stressful situation (meaning in terms of physical stress, for instance, illness or mental stress) and how they resolve these situations. The secondary outcomes of this study are the characteristics of the real lifestyle of our patients.

## 2. Methods

The study was conducted in accordance with the Declaration of Helsinki, and the project including its protocol was approved by a local ethical committee (e.g., Ethical Committee of University Hospital Motol, project No.15-25710A), and patients gave written informed consent for testing and using their data for further analysis before they participated in the study. Inclusion criteria were as follows: Confirmed T1D diagnosis with negligible residual endogenous insulin secretion (according to the presence of specific autoantibodies and C peptide levels), experience with real time CGM (RT-CGM) and no problems with skin tolerance during wearing CGM devices, no comorbidity that in itself or its treatment could interfere with glucose homeostasis (for instance, treatment with corticosteroids), a willingness to cooperate (perfect previous compliance), and completion of structured reeducation.

Thirty-five patients out of 37 patients who started the study were able to go through the complete analytic phase of the study. Of those 35 participants, 20 of them were CSII users and 15 were on the MDI regime. One patient did not complete the study because he was actively involved in martial arts and had a problem with glucose sensor tolerance. The second patient resigned from the study due to an unexpected opportunity to travel to an exotic destination for a longer period of time.

**Figure 1:**
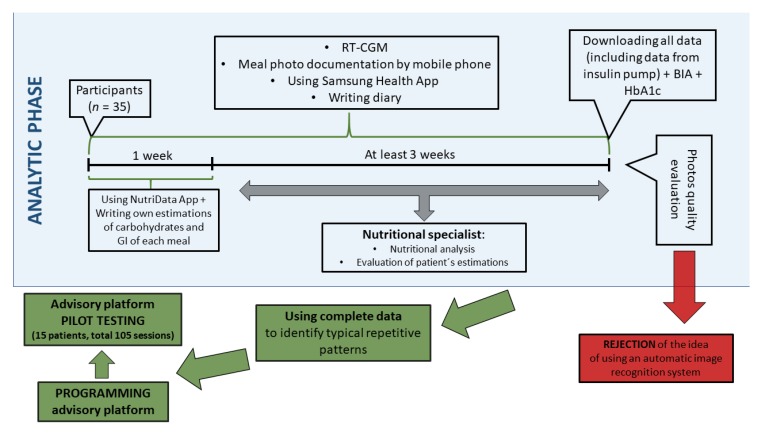
Study design. App—application; BIA—bioimpedance analysis; GI—glycaemic index; RT-CGM—real time continuous glucose monitoring.

All patients used insulin analogues. Patients on MDI and patients having an insulin pump other than Medtronic used as CGM system Dexcom G4 Platinum (*n* = 26). Patients using the Medtronic insulin pump (*n* = 9) had a Medtronic CGM system, which was directly linked to the pump. They were using the Enlite glucose sensors. Their basic characteristics were as follows: 19 females, 16 males; 16/35 suffered at least one chronic diabetic complication (the most frequent was retinopathy); 10/35 patients had multiple chronic diabetic complications (two and more); 9/35 patients had autoimmune thyroiditis (but were euthyroid at the time of the study); 6/25 patients were being treated for hypertension; and 7/25 used statins for dyslipidemia. Further clinical characteristics of the study group are shown in Table 1. Study protocol is summarized in Figure 1.

### 2.1. Nutritional Analysis, Patient’s Diary

For the duration of the study, patients were given mobile phones (Samsung Galaxy S5; OS Google Android 4.4 (Kitkat); camera 16MPx (4640 × 3480); Samsung, Seoul, South Korea) to photograph the food and monitor energy expenditure by using the Samsung Health application. Patients received detailed instructions on how to photograph the food (e.g., lay a ruler or a standard size item next to the food to serve as a measure, etc.). At the same time, patients were asked to keep a detailed diary, not only describing food, but also physical activity and other circumstances that affect glycemia (illness, other stress, menstruation, etc.). Patients treated with the MDI regimen also recorded insulin doses in their diary.

During the first week of the study, patients recorded consumed food also in the NutriData web application (https://nutridata.cz). For this purpose, they had to weigh foods, and in the case of complex meals, they had to describe specific recipes. At the same time, patients were asked to estimate the carbohydrate content of the meal according to their current habits and also to specify the glycemic index (whether glycemic index is low, medium, or high). Data from application NutriData were analyzed by a nutritionist using NutriPro Expert software (https://nutripro.cz) connected to a certified food database. Currently, patients are educated to calculate 10 g of carbohydrates as one CU (carbohydrate unit). Given that many of our patients have been treated since childhood, and pediatric diabetology in the Czech Republic had used a CU containing 12 g of carbohydrates for a long time before the unification with habits used in adult patients occurred, we considered the right answer to be the one that was within a range of 6 g of carbohydrates (representing one ½ of the “old” CU). This means that if, for example, a nutritionist has calculated that the meal contained 35 g of carbohydrates and the patient wrote 30, this result was considered correct. If the patient was mistaken by more than 6 but not more than 12 g carbohydrates, the result was marked as wrong. If the patient was mistaken by more than 12 g carbohydrates, this error was marked as a significant one. Note—patients did not use any dietary supplements or vitamins during the study.

### 2.2. Biochemical and Body Composition Analysis

The level of HbA1c was measured from a blood sample taken at the same time that a body composition analysis (using body impedance analysis BIA) was provided. At that time, patients were without signs of any acute illness, and all other chronic diseases (if present) were properly stabilized. BIA measurements were made during regular patient visits, using the body-composition device Bodystat Quadscan 4000 (Bodystat Ltd., Isle of Man, British Isles). Anthropometrical values (weight, height, waist, and hips) were obtained by a specially instructed nurse. Each patient was measured in a standard measurement scenario: Supine with electrodes attached to the right hand and foot. Each measurement was taken twice to exclude possible measurement bias. Data were obtained directly with BIAS version 6.0.1.394 software (Bodystat Ltd., Isle of Man, British Isles). For normal reference values, we used the manufacturer’s (Bodystat Ltd., Isle of Man, British Isles) reference data matched for the patient’s gender and age and results of a national anthropometric survey and recommendations of the Czech Diabetological Society (treatment target goals for T1D patients) [1,4]. BIA served not only to analyze the body composition of patients but also to determine the value of the basal metabolic rate for each patient.

### 2.3. System Architecture of the Advisory Platform

**Figure 2:**
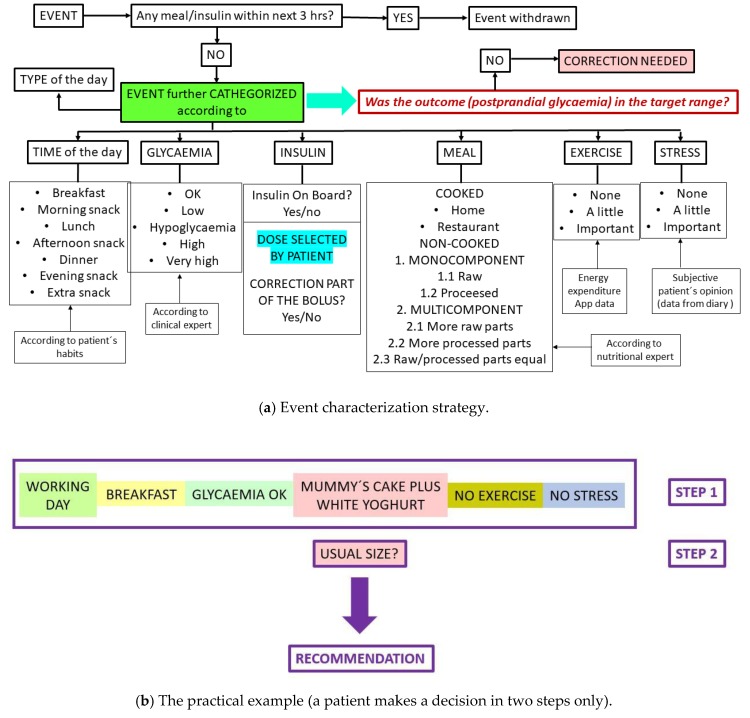
Event categorization. App—application.

In data obtained during the analytic phase, we tried to identify and categorize specific situations (events). To input key parameters (identifiers), the following data were used: Glucose levels (BG) from CGM records, insulin dose, food information, physical activity, stress, type of the day (working/not), and exact time of day. During the analytic phase of the study, when the specified situation (event) occurred at least three times, and if there were no modifying factors within the next 3 h (extra meal or extra insulin bolus, etc.), these data were used to choose the optimal insulin dose when the patient would encounter the similar (or very similar) situation again (Figure 2). To do so, we implemented several mathematical models, such as autoregressive models with exogenous inputs (ARX/ARMAX), a support vector machine for regression (SVR), and an extreme learning machine for regression (ELM). Additionally, we examined the hypothesis that a combination framework could perform better than any individual model; for that purpose, we applied a linear, a boost, and a bagging metaregressor. We also applied these models in combination with an optimization algorithm for computing the coefficients of the models. Models, compartment models, and evaluation criteria calculations were gathered using MATLAB R2016a software (MathWorks, Natick, MA, USA). This process was supervised by clinical experts. The prototype of the advisory application was programmed in JAVA and designed for OS Google Android.

### 2.4. Statistical Analysis

Data were statistically analyzed using SPSS sw.v.24 (IBM, Armonk, NY, USA) and checked for normality. Due to the data distribution, nonparametric tests were applied for group comparisons (the Mann-Whitney *U* test for the comparison of two groups) and for correlation analysis (Spearman’s correlation). The significance threshold was set at 0.05.

## 3. Results

### 3.1. Anthropometric Data

According to BMI readings, seventeen patients (49%) had an appropriate BMI, 34% of patients were overweight (*n* = 12), 17% were obese (*n* = 6), and no one was underweight. In women, being overweight/obese was more prevalent than in men (seven women were overweight, three obese). Moreover, in men, there were only two patients overweight/obese as confirmed by body fat content measurement using BIA; in other men having a BMI higher than normal, this finding was due to higher muscle mass. The anthropometric data of the patients are summarized in Table 1 and expressed separately for females and males.

### 3.2. Nutritional Analysis and Analysis of Eating Habits

Patients ate regularly with all eating at least three meals a day (mean number of meals per day was five, range was three to eight meals). In terms of energy content, lunch was the most important meal, which is in line with national habits. The second largest meal of the day was breakfast. No patients had other specific diet restrictions (such as for celiac disease), and none of the patients were on a low carbohydrate diet. Despite this, it was obvious that the intake of carbohydrates was frequently lower than recommended, with patients tending to reduce polysaccharides rather than simple sugars. In terms of macronutrients, we found the very high intake of saturated fat to be the biggest problem. The main source of saturated fat came from the consumption of chocolate bars, sweets, and biscuits as a “prevention of hypoglycemia.”

All the patients had lower than recommended fiber intake. The majority of the patients had suboptimal vitamin C intake as well. These results are not surprising because most of the patients ate few raw vegetables and fruits. On the other hand, sodium intake was mostly high (due to the frequent consumption of salty foods). Detailed results of the nutrition analysis are shown in Table 2.

Supplementary Table S1 shows how food was categorized for further analysis and the representation of each category. At least one meal a day was cooked, usually lunch. As for cooked meals, in 60% of such cases, it was a meal in a restaurant (canteen) and in the remaining 40%, home-prepared food.

### 3.3. Using Bolus Calculators

Use of a bolus calculator (BC) was unsatisfactory. All the patients treated by CSII had the pump with integrated BC, all had the necessary parameters preset by the diabetologist, and all were instructed how to use the BC. Despite this, only three patients out of 20 insulin pump users always used the BC, 9/20 patients occasionally (at least once per week) used the BC. Eight patients (six of them women) never used the BC. There was a strong association between BC frequency usage and diabetes duration, with less use of the BC by those patients who had diabetes for a longer time (*p* < 0.001, ρ = 0.56), but the connection to patient’s age was insignificant.

### 3.4. Accuracy of Patient’s Estimation of Carbohydrate Content and Glycemic Index

In the case of cooked meals, the occurrence of significant errors was 56%, and there was only 19% of the right estimation. A slightly better situation was in the case of noncooked meals, when significant errors were present in 44% of the cases, and the correct estimates were present in 36% of the cases. Women scored better than men (*p* = 0.02), but only in cooked meals.

Generally, the error rate increased with the amount of carbohydrates in the food evaluated (*p* = 0.01, ρ = 0.483).

On the other hand, the patients were quite able to precisely divide foods by their glycemic index, as 77% of their answers were correct.

### 3.5. Physical Activity, its Handling, and Solving Special Situations

Nine patients were actively engaged in sport activities, 10/35 patients participated in recreational sports. The remaining 16 patients did not participate in sports, and over the course of the follow-up, they did not exceed 6000 steps per day for more than 50% of the monitored days. We observed a strong correlation between a patient’s physical activity (expressed either as the number of steps or the number of active minutes per day) and the following factors: Patient’s sex: Women were less active (*p* = 0.042, ρ = −0.373), HbA1c: More active patients have a better HbA1c (*p* = 0.014, ρ = −0.445), and diabetes duration: Patients having diabetes longer were less active (*p* = 0.018, ρ = −0.437). Patients treated for thyroid gland disease were also less active (even were euthyroid at the time of the study, *p* = 0.011). Active patients consumed less fat content (*p* = 0.017, ρ = −0.433), but there was no significant correlation with either BMI or waist circumference.

During follow-up, active subjects (active and recreational athletes) experienced only occasional hypoglycemia (27% of such cases were marked by patients as “sport related,” the lowest glycemia value was 55 mg/dL). All active athletes addressed a higher energy expenditure with a lower dose of insulin (CSII-treated patients used a temporary basal dose, MDI-treated patients applied less insulin before a meal that preceded physical activity), and in 40% of “sport events”, patients also added fast-acting carbohydrates (juice, glucose bonbons). Rebound athletes (all on insulin pump therapy) used a temporary basal dose in 63% of the cases and in 65% of the events also added carbohydrates. Unfortunately, they often chose carbohydrates in the form of chocolate, chocolate bars, etc.

Seven CSII users and two patients on MDI therapy reported that during the study there was a situation that negatively affected them in glycemia. In seven cases, it was an illness with a fever, and in the remaining cases, the situation was described as psychological stress (university exam period and a car accident with no physical injury, respectively). In all cases, CSII patients applied correction boluses, but did not use the opportunity to solve the situation with changing a basal dose. MDI patients applied correction doses of rapidly acting analogue, but they adjusted accordingly basal insulin as well.

### 3.6. Sleeping and Working Hours

The patients’ average sleep time was 6.5 h, with one third of the patients reporting that for more than half of the nights, when they woke up in the morning, they felt a little tired and often woke up several times during the night. Glycemia, however, was rarely mentioned as a subjectively perceived cause of poor sleeping quality. We observed a significant correlation between the number of hours spent working (in terms of hours spent at work plus workload solutions at home, in leisure time) and time spent in hyperglycemia and glycemic variability (*p* = 0.001, ρ = 0.45, and *p* = 0.009, ρ = 0.407, respectively). It is necessary to emphasize that all the patients had rather sedentary jobs.

### 3.7. An Automatic Image-Recognition System

Patients took a total of 6893 meal photos during the monitored period. In 3% of cases, patients forgot to take a meal photo, but they put this information into their diaries. In spite of all the briefing, the quality of the photos was often poor, mainly because the photos were not well focused (this was the case in 29% of the photos, e.g., 1999 photos). Moreover, Supplementary Figure S1 shows that even if the quality of the photo was good, it was sometimes impossible to determine what food was in the photo without further specification.

For these reasons, we gave up on our original intention to use automatic recognition of the photography.

### 3.8. Pilot Results Using the Application Prototype

We selected breakfast to be the best model meal of the day as the most repetitions of categorized events were connected to this meal. Moreover, there were usually no other interfering factors, such as active insulin from previous boluses. On the other hand, breakfast was the most challenging meal for our idea as the highest occurrence of postprandial hyperglycemia was observed after it (due to frequent eating of cakes, etc.). In the verification phase of the prandial bolus optimization system, the patient had to select from the application menu only the type of event and confirm that the meal was of usual size (Figure 2b). Fifteen patients underwent this phase of the study. The patients had the choice to accept the advice or not. If the patients decided not to accept the advice, they were asked to record why they declined to accept the advice; however, the patients accepted all of them. One hundred and five such experimental sessions (all breakfasts in one week) were conducted, and in almost all the cases (95/105, e.g., 90%), postprandial glycemia was within target range (Table 3). By using this approach, the patients were able to reach postprandial glycaemia in the target range without calculations, so the patients evaluated the prototype application positively.

## 4. Discussion

Flexible insulin therapy (either CSII or MDI) allows the patient to lead a life without significant limitations. He or she can eat somewhat irregularly and according to his or her taste, but the patient must perform glycemic self-monitoring at a sufficient level (preferably with RT-CGM or FGM—flash glucose monitoring) and must be able to correctly calculate the carbohydrates [5].

We did not expect so many significant mistakes in carbohydrate counting in educated and cooperative patients. Our results clearly show that theoretical knowledge is one thing, and its successful application in real life is another. The second major problem that we encountered was the insufficient usage of a bolus calculator, but these two problems are closely interconnected. If patients used a bolus calculator, its recommendation was often incorrect due to basic input error (i.e., entering the wrong amount of carbohydrates). After several such experiences, the patients did not trust bolus calculator recommendations and used it less and less. According to our results, difficulty with carbohydrate counting was the biggest obstacle to reach postprandial normoglycemia.

Errors in determining the carbohydrate content in food is not just a problem for our patients. For example, the results of a 2016 study of 61 adult T1D patients found that only 59% of the participants were able to achieve a satisfactorily accurate determination of carbohydrate content [6]. A recently published study on adolescents found that carbohydrate counting is more of a problem for them than for adults, which is not surprising [7].

Regarding the inadequate use of BCs, one interesting finding was that longer-term patients have a more reserved approach to using it, but there was no connection with patient’s age. We believe this is because patients who have experienced a previously used approach (for example, less glycemic measurements) have greater difficulty adapting to a modern, highly technical-oriented treatment. It is clear that currently this is the only way to achieve tight diabetes compensation. In addition, technology can help patients solve problems effectively and in less time. However, some people simply do not trust modern technology and of course there are other patients who simply do not want to deal with diabetes, but this is primarily a psychological issue [8].

Our original idea of solving the problem with carbohydrate counting by using automatic food recognition in photos turned out to be technically unrealizable in the conditions of normal life. There are systems that provide dietary advice based on automatic carbohydrate counting, such as the GoCARB system, that have shown very promising results. This system, which is based on computer vision techniques, was originally tested for meals prepared in one concrete canteen [9]. Later on, an interesting analysis was provided when GoCARB estimates of the carbohydrate content of 54 central European plated meals were compared with estimates made by experienced dietitians. GoCARB and dietitians achieved comparable results, however, GoCARB had difficulty in estimating rice, pasta, potatoes, and mashed potatoes, while dietitians had problems with pasta, chips, rice, and polenta [10].

Our experience shows that this approach (automatic food recognition) would be applicable for commercially manufactured foods of known composition wherein a simple identifier could also be used by scanning the product’s barcode. The application needed for this scan could be installed into a mobile phone and linked to a validated food database, but these conditions are technically feasible. For meals prepared at home or for meals of generally unknown composition, another strategy would have to be employed.

We also confirmed findings made by Vasiloglou MF et al. that the larger the size of the meal and/or the higher the carbohydrate content of a food category, the more challenging it is to make an accurate estimation [10].

We found that with sufficient repetitions of the same/similar situation, an optimal dose of insulin can be identified and, in the case of insulin pump treatment, the optimal bolus form (for instance time and dose of the square component of a combined bolus). This approach is based on the classic learning process, with each attempt being improved by the incorporation of previous experiences. As early as 2010, a work focused on proposing an advisory treatment system was published, which compared rule-based reasoning and k-nearest neighbor algorithms [11]. Later calculators based on case-based reasoning (CBR) methodology were introduced. CBR may work with transforms that represent relations between analogical phenomena. In case of long-term monitoring of diabetic patients, it is possible to create a model of a particular patient and group different patterns (e.g., breakfast, lunch, dinner, etc. with typical meals and physiological conditions) having already known the outcome. Such a model can be successively used for more precise estimation/prediction of bolus for a given meal.

Recently an approach using artificial neural networks (ANNs) was developed but still the necessity of carbohydrate counting remains [12,13,14,15,16,17]. Moreover, according to our experience, algorithms that are trained on virtual subjects perform excellently on simulated data but perform much worse (or fail) on real data. According to our findings, the creation of a personalized (adapted to a particular patient data) and an easy-to-use application with a minimal number of parameter inputs is key. This would reduce the number of possible input errors and, at the same time, increase patient adherence.

The analysis of the patients’ lifestyles has revealed other fundamental issues that have the potential to further aggravate the health of our patients and their overall life prognosis, beyond time spent in hyperglycemia. We confirmed other recent studies regarding an increase in overweight and obesity in T1D patients [18]. This corresponds to our results from the analysis of the eating habits of our patients and their physical activity. Unhealthy eating (high fat and low fiber intake) is not a unique problem for our T1D patients [17]. For this reason, we plan to incorporate motivational elements into the next version of the application, which would naturally lead the patient to a healthier lifestyle.

Our study certainly has some limitations. The basic limitation is the study character, but we intended the study to be a proof-of-concept type so there is no formal power calculation due to a pilot study design. There may be some general arguments against our concept as well. For instance, this approach would not be suitable for patients with very irregular and unpredictable eating and daily regimes. The analytical phase of our proposal is rather complicated and time-consuming both for professionals and for patients, but for transfer to daily practice, it is certainly possible to simplify the process. Data from insulin pumps and CGM or glucometers are routinely downloaded at each check. More and more patients use CGM continuously (or almost continuously), so enough data exists for analytical algorithms. As far as nutritional analysis is concerned, this process can be significantly simplified for personalized application setup, with the nutritional diary in the form of photographs (which is easy and quick) and the food divided into groups without addressing its nutritional content. This will be necessary only if the patient is interested in knowing if his or her nutritional intake is well balanced.

## 5. Conclusions

Eating freely, according to personal taste and permanent normoglycemia, is currently still beyond the possibility of even the most advanced technologies. In the current and near future (before diabetes will, hopefully, one day be eradicated), our patients will still have to estimate the glycemic effect of their meals. To make it easier for them, we introduced an innovative strategy, with no carbohydrate counting, which was incorporated into the mobile phone application prototype. This strategy has emerged from the comprehensive analysis of many factors of the patients’ daily lives. Through that analysis, we also found out that our T1D patients live a very unhealthy lifestyle. A sedentary lifestyle and unhealthy eating present important problems for T1D patients, problems that must be solved to improve the life prognosis of these patients. These problems also should be addressed in new advisory (treatment) tools.

## Figures and Tables

**Table 1:** Group characteristics.

**General**	**Median**	**SD**	**Range**			
Age (years)	39	12	19–64			
Diabetes duration (years)	19	10	0–37			
CSII therapy duration (years)	7	7	1–27			
Experience with CGM (months)	8	18	2–60			
HbA1c (mmol/mol)	58	11	35–76			
HbA1c DCCT (%)	8	1	5–9			
Insulin dose per kg (IU/kg)	0.5	0.2	0.2–1			
Anthropometric and Body Composition Data	**Females**			**Males**		
	**Median**	**SD**	**Range**	**Median**	**SD**	**Range**
BMI	25	5	19–37	26	3	22–33
Waist (cm)	85	11	67–111	87	9	75–106
Hip (cm)	99	13	90–132	103	9	90–116
Fat (%)	30	7	19–41	14	6	7–24
ABM (%)	70	7	59–82	86	6	76–93
Water (%)	51	6	43–62	62	4	55–68
BMR (kcal/kg)	23	3	17–28	26	2	21–29

Note to Table 1, specifically to the continuous glucose monitoring (CGM) system using experience prior the beginning of the study: All patients had some practical experience of using real time CGM system before starting the study. Eighteen patients used real time CGM (RT-CGM) almost permanently before entering the study. In Table 1 there is specified the time how long these patients used RT-CGM at least 70% of the time prior the study. ABM: Active body mass; BMI: Body mass Index; BMR: Basal metabolic rate; DCCT: Diabetes control and complication trial; SD: Standard deviation.

**Table 2:** Nutrition analysis results in % of daily recommended intake; done by using NutriPro Expert software (Note: Patients did not use any dietary supplements and/or vitamins and this data format was chosen in order to compare patients with each other.).

**% of Daily Recommended Intake**	**Median**	**SD**	**Range**
**Energy**	97	31	62–138
**Carbohydrate**	78	31	68–147
**Sugars**	75	56	40–196
**Fat**	134	51	67–202
**Saturates**	162	54	100–233
**Protein**	156	44	87–223
**Fiber**	42	19	33–74
**Cholesterol**	70	103	37–300
**Calcium**	56	45	41–158
**Iron**	49	48	29–158
**Sodium**	118	54	100–214
**Potassium**	86	44	61–168
**Phosphor**	129	65	100–246
**Magnesium**	64	25	43–102
**Vitamin C**	37	25	19–84
**Distribution of Daily Energy Intake (%)**	**Median**	**SD**	**Range**
**Breakfast**	27	10	7–35
**Morning snack**	4	8	0–21
**Lunch**	36	5	28–41
**Afternoon snack**	12	8	3–25
**Dinner**	17	9	0–26
**Evening snack**	8	4	0–11

SD—standard deviation.

**Table 3:** Improving postprandial glycaemia when using the application.

	Time											
	6–7 am	6–7 am	7–8 am	7–8 am	8–9 am	8–9 am	9–10 am	9–10 am	10–11 am	10–11 am	11–12 am	11–12 am
% High (>180 mg/dL)	15	0	48	0	58	0	54	16	27	4	12	0
**% In Range** **(80–180 mg/dL)**	**85**	**92**	**48**	**100**	**42**	**98**	**46**	**82**	**73**	**96**	**88**	**100**
% Low (55–80 mg/dL)	0	8	5	0	0	2	0	1	0	0	0	0
% Urgent Low (˂55 mg/dL)	0	0	0	0	0	0	0	0	0	0	0	0

Involved are glycaemia data from 15 patients from all their breakfasts in one week without application followed by the week using the application, values are expressed as median.

## Supplementary Materials

The following are available online at https://www.mdpi.com/2072-6643/11/5/1148/s1, Figure S1: Even an experienced nutritional therapist could not perfectly pinpoint the food in the photo without having further information, Table S1: Food categorization.
