# Supplementary material for: Comprehensive Analysis of the Real Lifestyles of T1D Patients for the Purpose of Designing a Personalized Counselor for Prandial Insulin Dosing
Source: Nutrients. 2019 May 23;11(5):1148. doi: 10.3390/nu11051148 (PMC6567095; doi:10.3390/nu11051148)

Supplementary Table s1 Food cathegorization

	Monocomponent meals		Multicomponent meals		Cooked meal	
	Example: apple or yoghurt as a snack		Example: bread with butter, ham and sliced tomato		Example: roasted turkey with potatoes and carrots (RTPC)	
	**Raw**	**Processed**	**Processed**	**Raw**	**Home-made**	**Restaurant/ Canteen**
**Example**	Apple	Yogurt	Bread, butter, ham	Tomato	RTPC home-made	RTPC Canteen

Supplementary Figure S1: Even an experienced nutritional therapist could not perfectly pinpoint the food in the photo without having further information

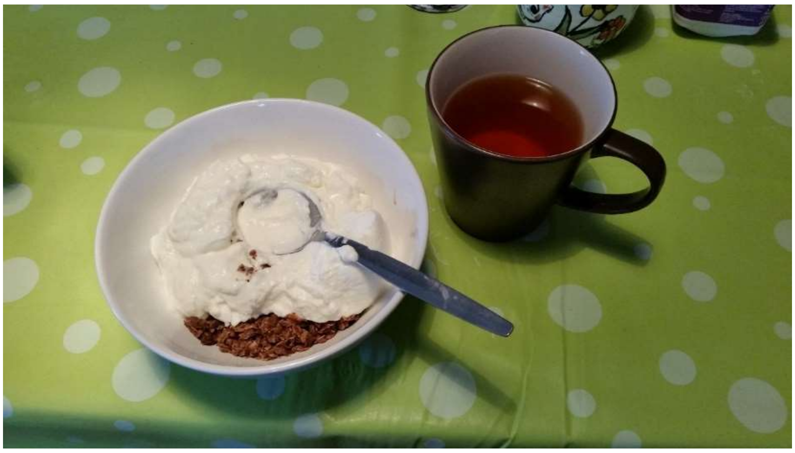

Supplement: Supplementary file 1 [file nutrients-11-01148-s001.pdf]
